# Nurses’ Knowledge toward Hepatitis B and Hepatitis C in Guilan, Iran

**DOI:** 10.2174/1874434601711010034

**Published:** 2017-04-17

**Authors:** Farahnaz Joukar, Fariborz Mansour-Ghanaei, Mohammad Reza Naghipour, Tolou Hasandokht

**Affiliations:** Gastrointestinal & Liver Diseases Research Center (GLDRC), Guilan University of Medical Sciences, Rasht, Iran.

**Keywords:** Nurse, Hepatitis B, Hepatitis C, Knowledge, WHO, HIV/AIDS

## Abstract

**Background:**

Health care workers (HCWs) represent high risk population for viral hepatitis infection.

**Objectives:**

This study sought to assess the knowledge of HCWs regarding hepatitis B (HBV) and hepatitis C (HCV) infection.

**Methods:**

In a multi-center cross sectional study, all HCWs from eight teaching hospitals were invited to participate in the study and to fill in a self-administered questionnaire.

**Results:**

A total of 1008 eligible HCWs have responded to the study. A high proportion of the study participants (55.4% and 52.9%) had unsatisfactory knowledge about HBV and HCV. Mean knowledge score toward HBV was significantly higher among more educated staff, p <0.001 and vaccinated personnel, P=0.02. Majority of responders answered correctly to transmission questions toward HBV and HCV (90% and 80%, respectively). There was statistically significant difference in only transmission domain score between various hospitals (p<0.05). The highest scores were related to surgical hospital.

**Conclusion:**

Although more than ninety percent of our participants were educated about HBV and HCV, knowledge about nature of disease, prevention, treatment and vaccine availability was unsatisfactory. Continuous training program toward viral infection is a matter of necessity.

## INTRODUCTION

Hepatitis B (HBV) and C (HCV) are two common, serious causes of chronic liver disease and liver damage. The World Health Organization (WHO) has reported that more than 500 million people are living with chronic viral hepatitis in the world [1, 2]. Since,50% to 80% of HCV infection leads to chronic hepatitis [3, 4], the increasing burden of HCV infection is considerable. In addition, an estimate of 57% of liver cirrhosis and 78% of liver cancer are considered due to hepatitis B virus (HBV) or hepatitis C virus (HCV) infections [1, 2]. The Middle East country and Iran located in the intermediate endemicity with a carrier rate between 2% and 8% [5]. Percutaneous injuries are one of the most common routes of HBV and HCV [6, 7]. According to the WHO, about three million individuals are injured per year due to needle stick or sharps injuries [8]. Health-care workers(HCWs) including nurses are potentially at high risk of blood-borne diseases such as HBV or HCV. Studies reported the risk of pathogens transmission HCWs by a needle stick injury (NSI) has been estimated to be between 6% and 30% for HBV and between 3% and 10% for HCV [9]. An average seroconversion rate for HCV in occupational exposure has been estimated 1.8% (range: 0-7%) [10]. Although NSI hepatitis seroconversion is somewhat rare, the costs of treatment and anxiety about the possible consequences of an exposure are serious. In this area, prevention through immunization and increasing knowledge are the safe strategy against the high prevalence of viral hepatitis among HCWs. For this reason, some HCWs refuse to service patients with blood born disease such as HIV/AIDS, HBV and HCV [11-14]. On the other hands, several studies reported the nurses who are informed about the mode of HBV and HCV transmission will have a positive attitude and confidence in treating or handling patients [11-15]. Hence, HCWs should familiarize themselves with guideline designed to prevent transmission of HBV, HCV and other blood-borne pathogens. The purpose of this study was to assess knowledge towards HBV and HCV among nurses in different hospitals of Guilan University of Medical Sciences, Rasht, Iran.

## MATERIALS AND METHODOLOGY

A questionnaire based cross- sectional study was conducted in various teaching hospitals setting of Rasht, the capital city of Guilan province, North of Iran. All study hospitals provide tertiary health-care level to a large population of Guilan province. All HCWs agreed to participate and filled in the questionnaire were included in the study. The participants were asked to complete a Self-report questionnaire separately under supervision of a trained research assistant. Sample enrollment, data gathering and data entry were supervised by a**research assistant.

We used the Richmond *et al.* [15] self report questionnaire with some modifications. In our previous research, the face and content validity of the developed and standardized questionnaire was determined by a panel of experts of Gastrointestinal and Liver Diseases Research Center (GLDRC) of Guilan University of Medical Science (GUMS) [7]. This specifically designed questionnaire has been reported to be acceptable to almost all responders in a pilot study, with a cronbach's alpha coefficient of 0.7 for HBV knowledge and alpha = 0.8 for HCV knowledge. The questionnaire consisted of 70 questions and 3 main parts (including 22 questions on demographic data, job category, HBsAb, vaccination status, family history of HCV or HBV and history of NSI, 26 questions on the knowledge about HBV and 22 about level of knowledge on HCV infection. Knowledge section for HBV and HCV included the following category focusing on :A) nature of disease, consisted of 5 statements B) modes of transmission consisted of 11 statements for HBV and 10 statements for HCV C) Ways of preventing HBV and HCV infection consisted of 5 statements and D) treatment of HBV and HCV consisted of 2 statements . Hepatitis B questionnaire had an additional three questions about the availability of vaccine. Knowledge questions were propositions to which true/false/do not know could be replied. The total knowledge score of the participants was calculated by giving one point to each correct answer with a maximum possible score of 26 for HBV and 22 for HCV and minimum score of zero. The aim of the study was explained to participants, and were asked namelessly responded to the questionnaire.

The study design and written informed consent (per the Helsinki declaration) were approved by the ethics committee of the gastrointestinal and liver diseases research center of Guilan University of Medical Sciences.

### Statistical Analysis

Mean knowledge score toward hepatitis B and C was reported. Final results were explained based on the different sex groups, work experience, education level, education of NSI, history of vaccination, having history of NSI and source of information. Also, the mean knowledge score was determined based on each demographic group. Total score less than mean was considered as unsatisfactory whereas higher than mean was considered as satisfactory knowledge. A P value of <0.05 was taken as significant for independent T test and one way ANOVA. Data were analyzed by Statistical Package for Social Sciences (SPSS) Version 16.0.

## RESULTS

### Sociodemographic Characteristics

A total of 1008 nurses from different hospitals were recruited for the study and response rate were 55%. The sociodemographic characteristics of the responders and non-responders were similar (p>0.05).

The majority of participants 947(93.9%) were female (p< 0.05) with the mean age 34.7 (8.2%) years old for women and 39.2 (8.1%) years old for men. More than 94.2% of responders had history of immunization. Fifty seven percent of nurses reported history of NSI and 946 (93.8%) receive education program focusing NSI. The major source of their information was from book/educational class (71.7%). (Table **1**).

### Demographic Characteristics and Mean Knowledge Scores

A significant difference was found between the nurses’ knowledge on HBV concerning the education level (P=0.001) and history of vaccination (P=0.02). As shown in (Table **1**), there was no statistically significant difference between nurses’ knowledge toward HCV and the sociodemographic variables.

### Assessment of Knowledge Towards Hepatitis B & C

In general, the mean knowledge score for the entire study subject toward HBV and HCV was 15.2(±2.5) and 12.5(±3.6) respectively. The scoring range and mean obtained score toward knowledge domains are shown in (Table **2**). Approximately 50 percent of responders obtained unsatisfactory score toward HBV and HCV (55.4% and 52.9%, respectively).

More than 80% of responders correctly answer to 2 questions related to the nature of HBV and HCV, 7 questions about mode of transmission of HBV and HCV, only two questions in area of the prevention strategy about HBV. More than 90% of responders knew that vaccine develop immunity toward HBV infection. Fortunately 81% of participants knew the correct answer about HBV vaccination schedule. (Table **2**).

### Demographic Characteristics and Knowledge Scores in Each Domain

As seen in (Table **3**), a significant difference was found between the nurses’ knowledge about transmission, vaccine, prevention and treatment toward HBV concerning the education level (P<0.005). There was a significant difference in mean score of transmission, availability of vaccine and treatment domains regarding history of vaccination (P<0.005). Also, statistically significant difference was seen between mean score of treatment domain and vaccine availability with work experience (P<0.005). The mean score about vaccine availability was significantly better in men than women (P<0.005).

The mean score of the nature of disease and treatment toward HCV were statistically significant different based on the history of education about NSI and sex group, respectively (P<0.005).

### Demographic Characteristics and Knowledge Scores in Eight Hospitals

Although, total knowledge score toward HBV and HCV between all eight various hospitals of Guilan University were relatively similar (p>0.05), there was a statistically significant difference in knowledge score in transmission domain of HBV and HCV (p<0.05). The highest scores in mode of transmission domain about HBV and HCV were related to Guilan surgical hospital (7.5±1.4) and internal medicine hospital (7.3±2) respectively.

## DISCUSSION

In the present study, among all study participants less than half had satisfactory knowledge about HBV and HCV. In the same way, in several study HCWs’ knowledge toward HBV and HCV were relatively low [16-20]. Majority of responders reported good knowledge about mode of transmission toward HBV and also HCV. A small percentage of respondents actually knew about nature of disease, prevention and treatment strategy. Our results are in line with the findings from studies reported majority of medical students and HCW had correct knowledge on transmission mode [16, 17, 21]. Yamazhan *et al.* in a multi-centre cross-sectional study showed that nursing students had a low level of knowledge about nature of the disease and its complications [22].

The most important source of information was through book and educational class and unsurprisingly HCWs with higher educational level were more aware about HBV. Responders’ knowledge score in all domains were higher than among advanced educational level. Our results are in line with the findings from studies reported more educated worker related with more knowledge toward HBV and HCV [15, 23, 24]. Setia *et al.* investigated awareness toward HBV and HCV amongst HCWs of a tertiary level hospital in India, indicated nursing students’ knowledge was considerably lower than the dental and medical students [24] As well as, a study in Turkey indicated that mean knowledge score was significantly higher in fourth-year students than third-year students [25].

Several studies tried to show the effect of HCWs training on knowledge to viral hepatitis, reported participants who received more education showed considerable improvement in their knowledge levels [6, 26, 27].Therefore, providing more education course can help health staff to remember the preventative measure.

In the current study, almost half of the HCWs had history of NSI whereas 93% of them pass educational course about NSI. Perhaps the reason for the such high rate of NSI in spite of passing education was carelessness because of high workload. Similarly, Joukar *et al.* in a survey showed 54% of health care professionals had a history of NSI [7]. In another study, nurses who had NSI history showed considerably higher knowledge scores toward HBV, while knowledge score concerning having NSI history in our study was not different.

We reported similarity in total knowledge score toward HBV concerning work experience, but more experienced worker mentioned better score in questions related vaccine availability and treatment strategy. Joukar *et al.* in their study declared the mean score between the work experience groups was similar [7]. This finding indicate the result of repeated educational course during work years. Abozead *et al.* reported the high frequency of NSIs among HCWs while, more than 60% of nurses did not pass any educational program about viral hepatitis immunity and control [28]. In the same way, Askarian *et al.* in a cross-sectional study revealed 71.1% of the students had NSIs, unfortunately 82% of them did not reported and worked up [29]. Our study participants who had history of immunization were more knowledgeable about HBV, especially in relation to mode of transmission, vaccine availability and treatment strategy. Responders with history of education about NSI showed the higher score about nature of HCV.

Our study revealed a similarity in knowledge score at the eight university teaching hospitals, and also, the mean score of the all eight hospital were less than mean total score. This finding may be due to insufficiency of educational program in the hospital setting especially after graduation. Awareness levels derived from previous survey were unsatisfactory in all HCWs even medical students. Despite, the majority of HCWs passed the educational program, they showed unsatisfactory knowledge on causative agents, mode of transmission, symptoms and prevention strategy. As well as, a high frequency of NSIs reported in health staff. Hence, extensive and effective continuing health educational course should be provided to health staff. Health staff should be sensitive and responsible for own health and wellbeing creating a culture of self-care among HCW, vaccine availability and accessibility may increase healthcare [7, 16, 17, 20, 22, 24, 30-41]. Juon *et al.* [42] in a randomized control trial evaluated the effectiveness of a community based education program in improving HBV knowledge among 877 Asian Americans. They found even a 30-minute educational program can increase the level of knowledge after 6-month follow-up. All previous study recommended extended health educational program and increase the quality of training, but there is a question whether such program can warranty the safety of health care workers.

In the present study, we had large sample sizes, which increase the statistical power. While majority of previous studies conducted in small sample size [7, 19, 34, 40] an important aspect of the current study refers to comparing knowledge in a multicenter survey between tertiary level hospitals of our University. As well as, we tried to review the results of the pervious study about knowledge of hospital staff. Our study, while having much strength, involved some limitations that should be considered [2]. Only individuals who agreed to complete survey were eligible for participation in the study. In fact we did not try to encourage non responder to participate. However, there was no significant difference in demographic characteristics between responders and non responders [1]. We did not coordinate a specific time and place for filling questionnaire, which might decrease the none response rate. Because the most reason for none responding was lack of time.

## CONCLUSION

The results of this study indicate a low level of knowledge among HCWs about the hepatitis B and hepatitis C, especially in nature of disease, prevention and treatment strategy. More educated worker reported better knowledge toward viral hepatitis. In spite of the majority of HCWs had NSI education, more than 50% of them reported history of NSI. In service training about universal and standard precaution and also improvement in work condition can decrease the probable risk for health professional.

## Figures and Tables

**Table 1:** Comparison of demographic characteristics and mean knowledge scores (N = 1008).

			**Mean (SD) knowledge score toward Hepatitis B**	P Value*	**Mean (SD) knowledge score toward Hepatitis C**	P Value*
		**N (%)**				
**Work experience (years)**	**< 5**	360(35.7)	14.9 ± 2.6	NS	12.4 ± 3.8	NS
	**5-10**	231(22.9)	12.3 ± 2.6		12.5 ± 3.5	
	**>10**	417(41.4)	15.4 ± 2.6		12.5 ± 3.3	
**Sex**	**Female**	947(93.9)	15.2 ±2.6	NS	12.5 ±3.6	NS
	**Male**	61(6.1)	15.4 ± 3.2		12.4 ± 3.1	
**Education**	**Diploma**	84 (8.3)	14.9 ± 2.4	0.001	12.7 ± 4.1	NS
	**Under graduate**	54 (5.4)	15.5 ± 3.2		12.3 ± 3	
	**Post graduate**	870 (86.3)	16.1 ± 2.6		12.7 ± 2.4	
**History of vaccination**	**Yes**	950(94.2)	15.2 ± 2.6	0.02	12.5 ± 3.5	NS
	**No**	58 (5.8)	16.1 ± 2.7		12.4 ± 4.4	
**Education of NSI**	**Yes**	946 (93.8)	15.3 ± 2.6	NS	12.5 ± 3.5	NS
	**No**	62 (6.2)	15.1 ± 3.4		12.2 ± 4.1	
**History of NSI**	**Yes**	578 (57.3)	15.2 ± 2.6	NS	12.5 ± 3.6	NS
	**No**	430 (46.7)	15.3 ± 2.6		12.6 ± 3.5	
**Information**	**Book and Educate**	724(71.7)	15.1± 2.6	NS	12.5 ± 3.3	NS
	**Internet and TV**	31(3.1)	14.7 ± 2.3		12.5 ± 2.5	
	**Family**	41(4.1)	15.3 ± 3.2		11.9 ± 4.2	
	**Combine**	212(21.1)	15.3 ± 2.2		12.7 ± 3.2	

Independent T test and one way ANOVA, p<0.05.

**Table 2:** Responses to hepatitis B and C knowledge items.

Domain	HCV 12.5±3.6	HBV 15.2±2.5	Statement	**Correct answers in 1008 of responders**	
				HBV **N(%)**	HCV **N(%)**
Nature of the disease	µ = 2.8 ± 0.9	µ = 2.9 ± 0.8	Hepatitis B/C is a bacterial disease.	117 (11.6)	106 (10.5)
			Hepatitis B/C is a Contagious disease.	801 (79.3)	791 (78.3)
			Hepatitis B/C can lead to cirrhosis	925 (91.6)	862 (85.5)
			Hepatitis B/C is associated with an increased risk of liver cancer	876 (86.7)	853 (84.5)
			Once you have had hepatitis B/ C, you cannot catch it again because you are immune	227 (22.5)	183 (18.1)
Transmission	µ = 6.9 ± 2.2	µ = 7.3 ± 1.2	Hepatitis B can be spread through close personal contact such as kissing or talking	140 (13.9)	--------------
			Hepatitis B/ C can be spread through sharing injecting equipment, such as needles and operation tools	982 (97.2)	892 (88.3)
			Hepatitis B/ C can be transferred from mother to fetus	948 (93.9)	873 (86.4)
			Hepatitis B/ C can be spread by mosquitoes	266 (26.3)	262 (25.9)
			Hepatitis B/ C is spread through blood-to-blood contact	967 (95.7)	887 (87.8)
			Having a medical and/or dental procedure increases a person’s chances of contracting hepatitis B/C	956 (94.7)	904 (89.5)
			Hepatitis B/ C is spread through the air in an enclosed environment (e.g., crowded buses and elevators)	54 (5.3)	143 (14.2)
			Sexual transmission is a common way hepatitis B/ C is spread	922 (91.3)	824 (81.6)
			. Some people with hepatitis B/ C were infected through unsterile tattooing	975 (96.5)	900 (89.1)
			Some people with hepatitis B/ C were infected through blood transfusions	971 (96.1)	897 (88.8)
			Hepatitis B/ C Can be transmitted by sharing dishes	249 (24.7)	360 (35.6)
Prevention	µ = 1.9 ± 1.3	µ = 2.5 ± 0.9	HBV/ HCV can be prevent by vaccine	932 (92.3)	355 (35.1)
			HBV/ HCV can be prevent by regular exercise	59 (5.8)	90 (8.9)
			HBV/ HCV can be prevent by healthy diet	86 (8.5)	122 (12.1)
			HBV/ HCV can be prevent by hand washing	574 (56.8)	591 (58.5)
			HBV/ HCV can be prevent by condom	902 (89.3)	812 (80.4)
Treatment	µ = 0.8 ± 0.6	µ = 1 ± 0.7	There is a pharmaceutical treatment available for hepatitis B/ hepatitis C	345 (34.2)	454 (45)
			Special diet is recommended for patients with Hepatitis B/ hepatitis C	584 (57.8)	386 (36.4)
Vaccine	-------	µ = 1.5 ± 0.7	HBV vaccine cause immunity	911 (90.2)	-------------
			Pregnant women vaccination can be prevent fetus infection	414 (41)	-----------------
			Hepatitis B vaccine is given as 2 shots	201 (19.9)	--------------------

**Table 3:** Comparison of demographic characteristics and mean knowledge scores in each domain.

		HBV (Mean ± Std)						HCV (Mean ± Std)				
Domain and number of statements		Nature of the disease(5)	Transmission(11)	Prevention(5)	Vaccine(3)	Treatment(2)	Total (26)	Nature of the disease(5)	Transmission(10)	Prevention(5)	Treatment(2)	Total (22)
Experience(years)	< 5	2.9±0.9	7.3 ± 1.3	2.5 ±0.9	1.4 ± 0.6*	0.9 ± 0.6*	14.9 ± 2.6	2.1 ± 1	6.8 ± 2.6	2.1± 1.1	0.9 ±0.7	12.4 ± 3.8
	5-10	2.9±0.5	7.4±1.3	2.5±0.8	1.5±0.7*	1±0.7*	12.3 ± 2.6	2.8±0.1	6.9±1.9	1.9±1	0.8±0.7	12.5 ± 3.5
	>10	2.9±0.8	7.4±1.9	2.5±0.8	1.6±0.7*	0.9±0.7*	15.4 ± 2.6	2.9±0.9	7.1±2	1.9±1.5	0.8±0.8	12.5 ± 3.3
sex	Female	2.9 ±0.8	7.4 ± 1.2	2.5 ± 0.8	1.5±0.7*	0.9 ± 0.6	15.2 ±2.6	2.8±0.9	6.9±2.3	1.9±1.3	0.9±0.6*	12.5 ±3.6
	Male	2.9±0.9	7.4±1.5	2.4±0.9	1.8±0.7*	0.8±0.7	15.4 ± 3.2	2.6±1.2	7.1±1.7	2.1±1.1	0.7±0.5*	12.4 ± 3.1
Education	Diploma	2.8± 0.9	7.7±1.2*	2.8±0.9*	1.8±0.8*	1.1±0.7*	16.1 ± 2.6*	2.8±1.2	7.1±2.1	1.9±1.3	0.8±0.7	12.7 ± 4.1
	Under graduate	2.9±0.9	7.3±1.4*	2.4±0.8*	1.5±0.8*	0.7±0.6*	14.9 ± 2.4*	2.8±1.1	7.1±1.1	1.7±1.1	0.8±0.7	12.3 ± 3
	Post graduate	2.9±0.8	7.3±1.2*	2.5±0.8*	1.5±0.7*	0.9±0.7*	15.5 ± 3.2*	2.8±0.9	6.9±2.3	1.9±1.3	0.8±0.7	12.7 ± 2.4
History of vaccination	Yes	2.9±0.8	7.3±1.3*	2.5±0.8	1.5±0.7*	0.9±0.7*	15.2 ± 2.6*	2.8±0.8	6.9±2.3	1.9±1.3	0.8±0.7	12.5 ± 3.5
	No	2.7±1	7.7±1*	2.6±0.8	1.8±0.7*	1.1±0.7*	16.1 ± 2.7*	2.7±1.3	6.7±2.4	2.1±1.3	0.9±0.7	12.4 ± 4.4
Education of NSI	Yes	2.9±0.8	7.4±1.2	2.5±0.8	1.5±0.7	0.9±0.7	15.3 ± 2.6	2.8±0.9*	6.9±2.3	1.9±1.3	0.8±0.7	12.5 ± 3.5
	No	2.8±0.8	7.2±1.7	2.7±0.9	1.5±0.9	0.9±0.7	15.1 ± 3.4	2.5±1.1*	6.9±2.2	2.1±1.2	0.7±0.6	12.2 ± 4.1
History of NSI	Yes	2.9±0.8	7.3±1.3	2.5±0.8	1.5 ±0.7	0.9±0.7	15.2 ± 2.6	2.7±0.9	6.9±2.4	1.9±1.5	0.8±0.7	12.5 ± 3.6
	No	2.9±0.8	7.4±1.2	2.5±0.8	1.5±0.7	0.9±0.7	15.3 ± 2.6	2.8±1.1	6.9±2.1	2±1.1	0.8±0.7	12.6 ± 3.5
Information	Book and Educate	2.9±0.9	7.4±1.2	2.5±0.9	1.5±0.7	0.9±0.7	15.1± 2.6	2.8±0.9	6.8±1.9	2.1±1.1	0.8±0.7	12.5 ± 3.3
	Internet and TV	2.8±0.7	7.2±1.3	2.2±0.8	1.3±0.7	0.8±0.6	14.7 ± 2.3	2.6±1.1	6.5±1.9	1.7±0.8	0.9±0.6	12.5 ± 2.5
	Family	3.5±0.8	7.4±1.4	2.5±0.8	1.5±0.7	0.9±0.6	15.3 ± 3.2	2.8±0.7	6.8±2	1.8±1.1	0.8±0.6	11.9 ± 4.2
	Combine	2.9±0.7	7.2±1.3	2.6±0.7	1.6 ±0.6	1±0.6	15.3 ± 2.2	2.8±0.9	7.1±1.6	2.1±1.8	0.9±0.6	12.7 ± 3.2

* Independent T test and one way ANOVA, p<0.05
